# A new method for accurate localization of the LV pacing lead from fluoroscopy images to MRI images: application to studies involving lead placement and CRT

**DOI:** 10.1186/1532-429X-16-S1-P141

**Published:** 2014-01-16

**Authors:** Jonathan D Suever, Gregory Hartlage, Stephanie Clement-Guinaudeau, Michael Lloyd, John Oshinski

**Affiliations:** 1 Wallace H. Coulter Department of Biomedical Engineering, Georgia Institute of Technology/Emory University, Atlanta, Georgia, USA; 2 Department of Radiology and Imaging Science, Emory University School of Medicine, Atlanta, Georgia, USA; 3 Department of Medicine (Cardiology), Emory University School of Medicine, Atlanta, Georgia, USA

## Background

In Cardiac Resynchronization Therapy (CRT), studies examining the importance of placing the LV pacing lead in the latest contracting segment have produced conflicting results. Some studies have shown higher response rates when the lead is placed in the latest contracting segment, while others show no relationship between lead placement and response. *All of these studies relied on subjective manual methods to retrospectively transfer the LV lead position from the fluoroscopy images to the wall motion data*. We have developed a new, objective method to map LV lead location onto the pre-implant MRI data using standard intra-procedural fluoroscopic imaging. The method was validated on a cardiac phantom and compared to manual methods.

## Methods

MRI: 3D MR coronary vein (MRCV) scans were performed using slow infusion of Gadolinium contrast agent. The coronary veins were identified manually throughout the imaging volume. Fluoroscopy: During CRT device implantation, dual-plane *venograms *were acquired (30° LAO and RAO). After LV lead implantation, dual-plane *lead location *images were acquired with the same orientations as the venograms, and the LV lead tip location was mapped onto the corresponding dual-plane venograms. Registration: The 3D MR coronary vein locations were back-projected onto the venograms, and corresponding branch points were used to determine the spatial relationship between each of the venograms and the MR imaging system. Using this relationship, the two lead localizers were used to project the lead position into the MR coordinate system, and subsequently displayed on the AHA 17-segment model. Phantom: The accuracy of the lead mapping was determined using a coronary vein phantom with six potential lead location sites marked with fiducials visible in both MR and fluoroscopy imaging (to determine *true *position). The accuracy of the lead registration technique was compared to the *true *lead position and to Mortensen's o'clock, a commonly employed manual technique for estimating LV lead position in CRT.

## Results

The *true *lead position and the lead position determined by our method were within the same AHA segment in all six sites within the phantom. The average circumferential error was 7 ± 4% of an AHA segment (Range: 2-11%) and the average longitudinal error was 13 ± 17% of an AHA segment (Range: 4-27%). Using Mortensen's o'clock method, the correct AHA segment was selected in only two of six sites (33%). The average circumferential error was 38 ± 17% of an AHA segment (Range: 7-54%) and the average longitudinal error was 58 ± 30% of an AHA segment (Range: 6-83%).

## Conclusions

LV lead registration using the 3D projection of lead location from dual-plane fluoroscopic images onto 3D coronary vein anatomy allows for more accurate localization of the LV pacing lead compared to manual methods. This method can be used to accurately register LV lead position to MR wall motion data, and enable assessment of the relationship between LV lead position and the site of latest contraction.

## Funding

This study was funded in part by the Graduate Research Fellowship Program from the National Science Foundation.

**Figure 1:**
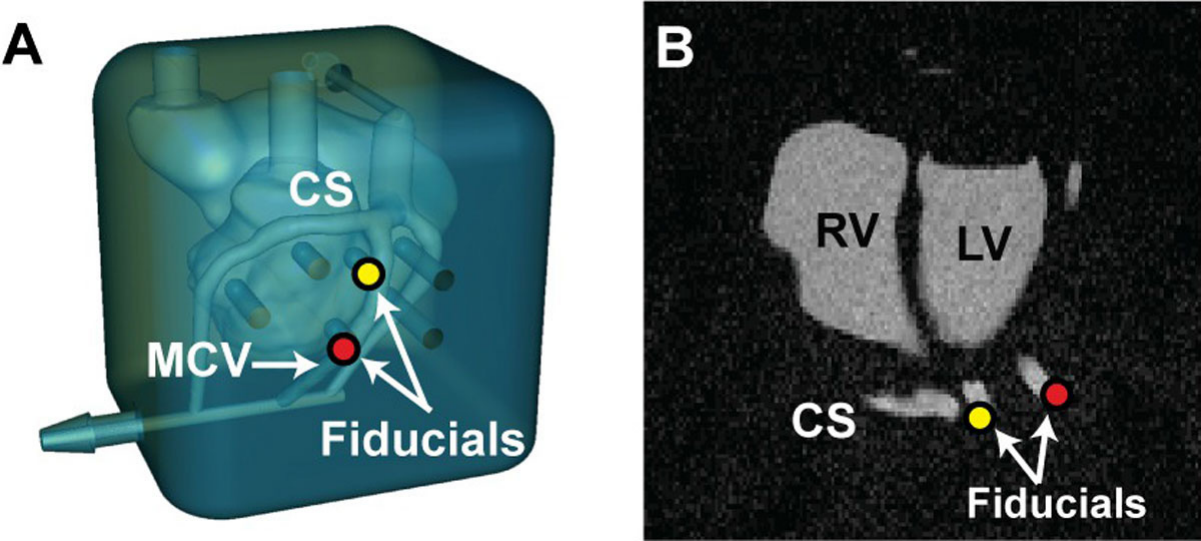
**Coronary Vein Phantom: A coronary vein phantom (A) was constructed with potential pacing sites (fiducials) that could be visualized with both x-ray and MR imaging (B)**.

**Figure 2:**
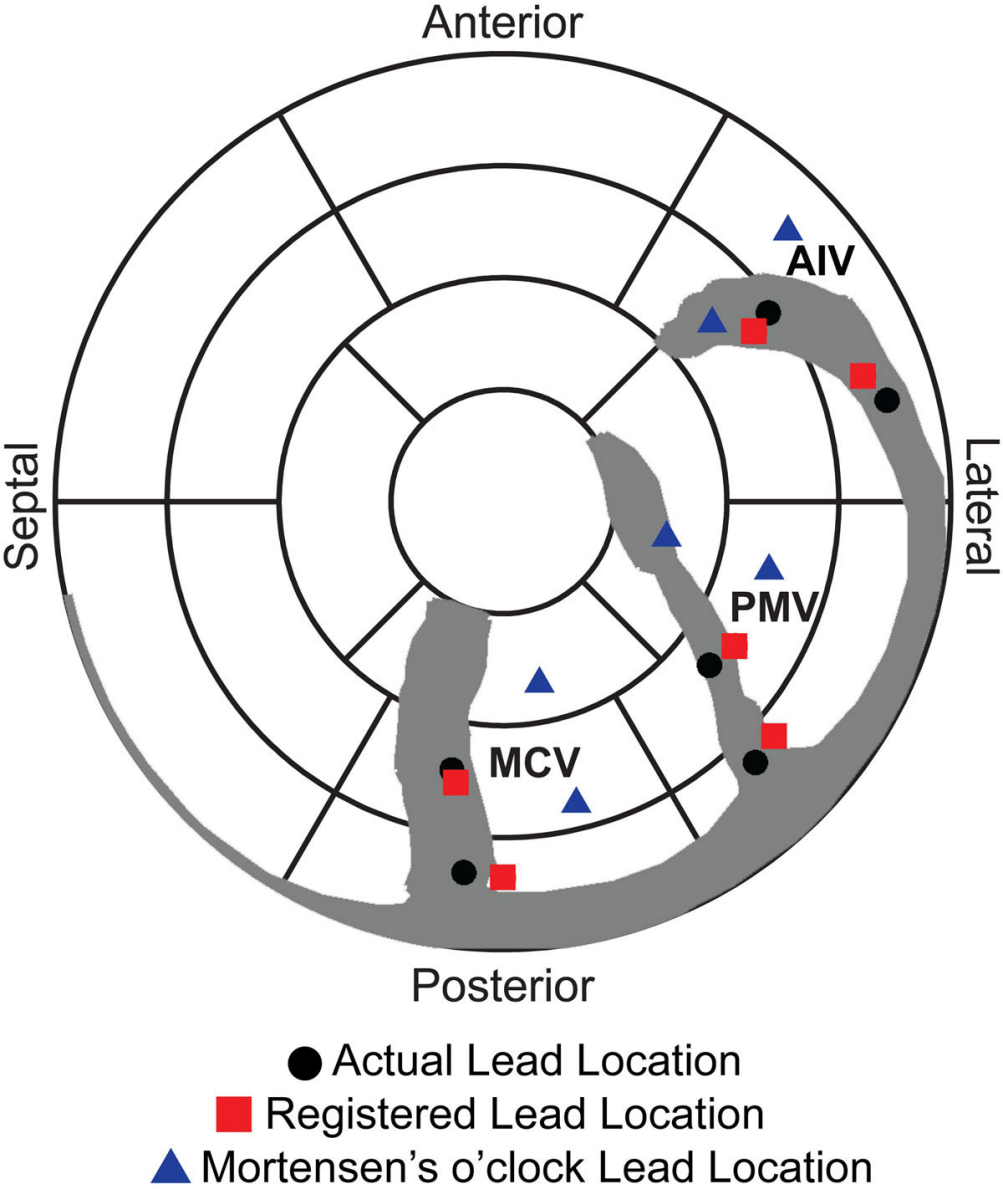
**Lead position in the phantom**. True lead locations imaged with MRI (black), LV lead locations mapped using our methodology (red), and LV lead location determined by Mortensen's o'clock (blue). Very small error was seen with our method, significant error was seen with the manual method, often locating the lead in the wrong segment.

